# [Retracted] Peritumoral activated hepatic stellate cells are associated with hepatic recurrence for resectable colorectal adenocarcinoma liver metastasis following resection

**DOI:** 10.3892/ol.2025.15362

**Published:** 2025-10-27

**Authors:** Li Deng, Taiyuan Li, Yuanyuan Liao, Shuang Liu, Zhen Xie, Zhixiang Huang, Hua Dai, Jianfeng Li, Xiong Lei

Oncol Lett 20: 287, 2020; DOI: 10.3892/ol.2020.12150

Following the publication of the above paper, it was drawn to the Editor's attention by a concerned reader that, concerning the immunohistochemical data shown in Fig. 2A on p. 7, the ‘NLT’ data panel was strikingly similar to a data panel featured in an earlier publication in the journal *Oncotarget* (albeit the contrasts of the images were different) written by largely different authors (with the possible exception of the first-named author of the article in *Oncotarget* paper, even though the named research institutes were also different).

Owing to the fact that the contentious data in the above article had already been published prior to its submission to *Oncology Letters*, the Editor has decided that this paper should be retracted from the Journal. The authors were asked for an explanation to account for these concerns, but the Editorial Office did not receive a reply. The Editor apologizes to the readership for any inconvenience caused.

